# Brachymetatarsia as an Early Clue to Turner Syndrome

**DOI:** 10.1002/ccr3.72365

**Published:** 2026-04-01

**Authors:** Hounaida Mahfoud, Zaki Elhanchi

**Affiliations:** ^1^ Gynecology, Obstetrics and Endocrinology Department Maternity Souissi, Ibn Sina University Hospital Rabat Morocco

**Keywords:** Brachydactyly, primary amenorrhea, skeletal malformation, Turner syndrome

## Abstract

Congenital anomalies of the extremities, particularly bilateral toe or finger malformations, may provide an early and valuable clue to an underlying genetic disorder and should prompt further diagnostic evaluation. Brachymetatarsia is a rare associated finding that may raise suspicion for Turner syndrome, particularly when diagnosis is delayed despite visible congenital anomalies.

## 1 Case Presentation

A 21‐year‐old woman presented to the gynecology department for primary amenorrhea. She had no notable medical or surgical history. She was a twin; her sister—non‐identical—had normal pubertal development and menarche at 12 years.

On clinical examination, the patient's height was 150 cm, and her pubertal development was Tanner S3 for breast development and P3 for pubic hair. External genital examination revealed a normal vulva.

**FIGURE 1:**
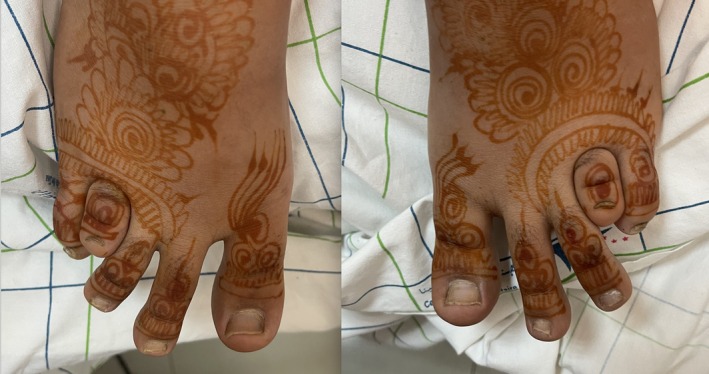
Congenital bilateral brachymetatarsia of the fourth toe.

During the general physical examination, a detail drew particular attention: bilateral brachymetatarsia of the fourth toe (type E Brachydactyly), present since birth but never investigated (Figure 1). Her twin sister did not share this anomaly. The hands were normal.

Pelvic ultrasound demonstrated a hypoplastic uterus with non‐visualization of the ovaries. Pelvic MRI further confirmed a hypoplastic uterus (UIb according to COVO classification), absence of identifiable ovarian tissue, and incidentally revealed a horseshoe kidney, an anomaly frequently associated with chromosomal disorders. Laboratory evaluation showed markedly elevated FSH with low estradiol levels, consistent with hypergonadotropic hypogonadism and ovarian insufficiency. Given the combination of primary amenorrhea, short stature, and uterine hypoplasia, cytogenetic analysis standard conventional GTG‐banded karyotyping on peripheral blood lymphocytes was performed and confirmed a non‐mosaic 45,X karyotype, consistent with Turner syndrome.

The patient was immediately started on hormone replacement therapy and referred for multidisciplinary follow‐up with endocrinology and gynecology for long‐term management.

## 2 Discussion

Skeletal malformations may represent an early diagnostic clue in girls with Turner syndrome [1]. While short stature, scoliosis, and congenital hip dysplasia are well‐described orthopedic manifestations requiring specialized follow‐up, subtle extremity anomalies can be equally important and may lead to an earlier diagnosis if recognized. Turner syndrome is associated with a broad spectrum of anatomical and physiological abnormalities, and its phenotypic expression is highly variable [2]. Although the condition is most commonly diagnosed around adolescence—typically around 13 years of age—when primary amenorrhea and hypergonadotropic hypogonadism become evident, earlier signs are frequently present but overlooked [3].

Among these early signs, brachymetatarsia, also known as type E brachydactyly (OMIM#113300), defined as an abnormally shortened toe (most often the fourth or fifth), is particularly relevant [2]. Approximately 20% of patients with Turner syndrome exhibit digital anomalies of the hands or feet [3]. Brachymetatarsia may be congenital or associated with systemic diseases such as Turner syndrome, Down syndrome, pseudohypoparathyroidism, Apert syndrome, and various endocrinopathies. In many cases, especially in TS, the anomaly is mild and discovered incidentally. Clinically, brachymetatarsia is most often a cosmetic concern and only occasionally causes discomfort due to mechanical imbalance of the forefoot [2].

In the context of Turner syndrome, a seemingly benign toe anomaly may represent a rare associated finding and should raise suspicion when accompanied by other suggestive clinical features. Careful examination of the extremities—particularly in newborns and young children—may prompt earlier genetic testing, allowing timely initiation of hormone therapy, appropriate screening for associated anomalies, and improved long‐term outcomes. In our patient, bilateral fourth‐toe brachymetatarsia was present since birth but remained unrecognized, contributing to delayed diagnosis of Turner syndrome until adulthood.

## Author Contributions

**Hounaida Mahfoud:** conceptualization, investigation, writing – original draft. **Zaki Elhanchi:** supervision, writing – review and editing.

## Funding

The authors have nothing to report.

## Consent

Written informed consent was obtained from the patient to publish this report in accordance with the journal's patient consent policy.

## Conflicts of Interest

The authors declare no conflicts of interest.

## Data Availability

The data that support the findings of this study are available from the corresponding author upon reasonable request.
